# Novel Mechanisms of Anthracycline-Induced Cardiovascular Toxicity: A Focus on Thrombosis, Cardiac Atrophy, and Programmed Cell Death

**DOI:** 10.3389/fcvm.2021.817977

**Published:** 2022-01-17

**Authors:** Silvio Antoniak, Sukanya Phungphong, Zhaokang Cheng, Brian C. Jensen

**Affiliations:** ^1^Department of Pathology and Laboratory Medicine, University of North Carolina School of Medicine, Chapel Hill, NC, United States; ^2^Blood Research Center, University of North Carolina School of Medicine, Chapel Hill, NC, United States; ^3^Department of Pharmaceutical Sciences, Washington State University, Spokane, WA, United States; ^4^Cardiology Division, Department of Medicine, University of North Carolina School of Medicine, Chapel Hill, NC, United States; ^5^Department of Pharmacology, University of North Carolina School of Medicine, Chapel Hill, NC, United States; ^6^McAllister Heart Institute, University of North Carolina School of Medicine, Chapel Hill, NC, United States

**Keywords:** anthracycline cardiotoxicity, thrombosis, myocardial atrophy, programmed cell death, protease activated receptor, FOXO1 (forkhead box O1)

## Abstract

Anthracycline antineoplastic agents such as doxorubicin are widely used and highly effective component of adjuvant chemotherapy for breast cancer and curative regimens for lymphomas, leukemias, and sarcomas. The primary dose-limiting adverse effect of anthracyclines is cardiotoxicity that typically manifests as cardiomyopathy and can progress to the potentially fatal clinical syndrome of heart failure. Decades of pre-clinical research have explicated the complex and multifaceted mechanisms of anthracycline-induced cardiotoxicity. It is well-established that oxidative stress contributes to the pathobiology and recent work has elucidated important central roles for direct mitochondrial injury and iron overload. Here we focus instead on emerging aspects of anthracycline-induced cardiotoxicity that may have received less attention in other recent reviews: thrombosis, myocardial atrophy, and non-apoptotic programmed cell death.

## Introduction

Considerable research effort has been invested in understanding the complex and multifactorial mechanisms underlying anthracycline-induced cardiotoxicity. Longstanding evidence has established causative roles for oxidative stress in contributing to cardiomyocyte dysfunction and death (1). Mitochondrial dysfunction generates much of this oxidative stress and the central role of multifaceted mitochondrial injury in anthracycline-induced cardiotoxicity has been comprehensively reviewed recently (2). Here, we will focus on emerging, though less-studied, mechanisms underlying the adverse effects of anthracyclines on both the heart and the vasculature.

## Anthracyclines and Thrombosis

Observational data suggest that some anti-cancer therapies are associated with increased risk for thrombotic events in the venous and arterial vasculature including deep vein thrombosis (DVT), pulmonary embolism (PE), and arterial thrombosis (AT) as recently summarized by Grover et al. (3). Indeed, Weiss et al. reported that 5% of stage II breast cancer patients (22/443) with 2 years of post-mastectomy chemotherapy developed venous thrombosis without signs of metastasis (4). Interestingly, no thrombosis was observed after completion of the chemotherapy (4). In another study of Stage IV breast cancer patients, thrombosis incidence rose to 17.6% in those who received anthracyclines (5). Interestingly, analysis of common risk factors for thrombosis (ambulatory status, obesity, family history, smoking, diabetes mellitus, hypertension, liver dysfunction, thrombocytosis, and previous endocrine therapy) showed no association with the observed thrombotic events (5). With specific regard to anthracyclines, multiple myeloma patients were at an increased risk of DVT (16%) when doxorubicin (DOX) was added to thalidomide and that risk increased with age (6). Importantly, the thrombotic risk for all three of these trials is reported relative to a control group that did not receive an anthracycline. Increased thrombosis incidence (7.5%) was also observed in breast cancer patients undergoing an anthracycline-containing chemotherapy regimen with age-dependent risk increase (27%) in patients over 60 years, though this study did not include a control group that was not exposed to anthracyclines (7).

Patient-specific factors that enhance risk of anthracycline-induced thrombosis are poorly defined, though one intriguing possibility is the metabolic syndrome. Individuals with the metabolic syndrome are at higher risk of both thrombotic events (8), and anthracycline-induced cardiotoxicity (9), possibly as a result of the chronically proinflammatory systemic milieu. Obesity (10) and insulin resistance (11, 12) components of the metabolic syndrome, also independently enhance risk for anthracycline-induced cardiotoxicity, though a direct link to thrombosis has not been established.

## Pro-thrombotic Effects on Vascular Cells

**Figure 1:**
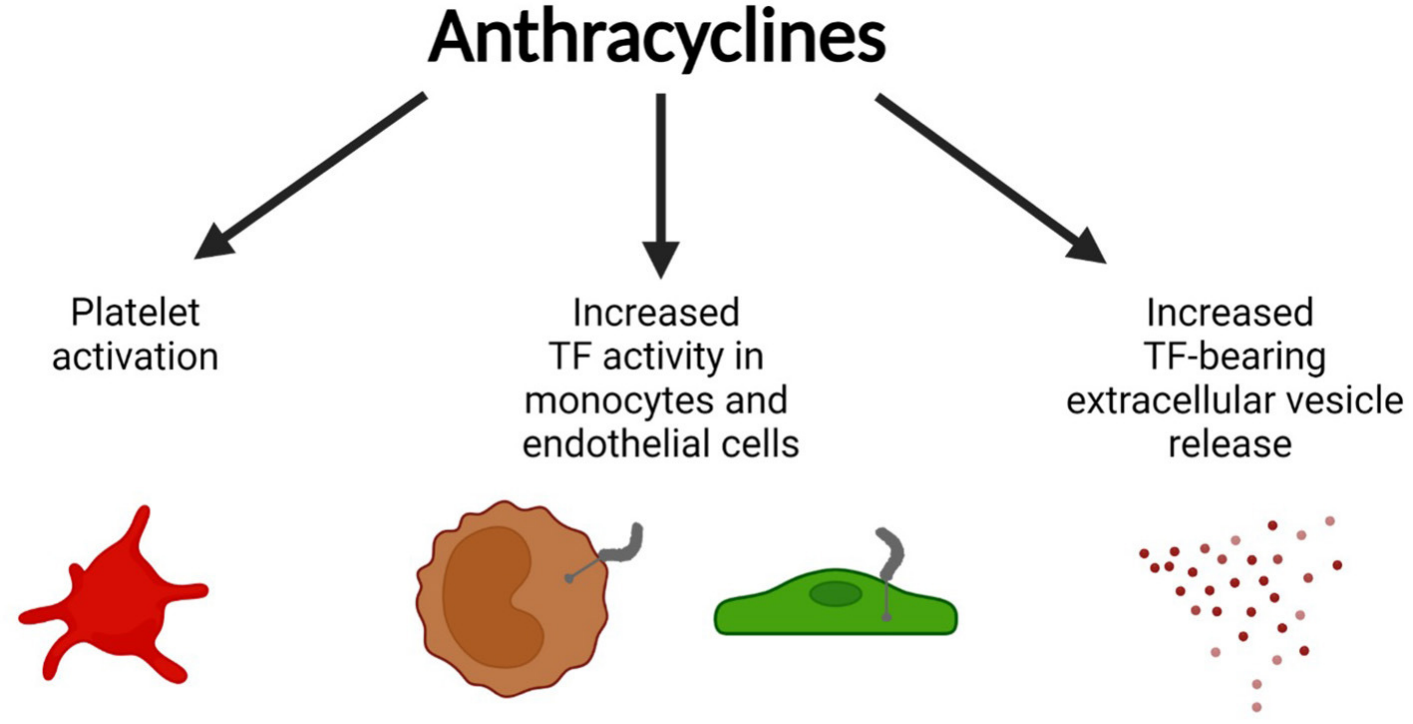
Prothrombotic effects of anthracyclines. Anthracyclines (doxorubicin, daunorubicin) activate vascular cells including platelets, monocytes, and endothelial cells leading to surface phosphatidylserine (PS) exposure, increased activity of pre-existing tissue factor (TF) on monocytes and endothelial cells, and the release of TF-bearing extracellular vesicles (EV). Figure created with BioRender.com.

How do anthracyclines, such as DOX, contribute to a prothrombotic phenotype? Multiple studies have shown that anthracyclines increase phosphatidylserine (PS) exposure on the outer cell surface on vascular cells (13–16). Negatively charged PS-rich membranes enhance the coagulation cascade reaction by increasing the activity of gamma carboxyglutamic acid (GLA)-dependent coagulation factors like factor VIIa (FVIIa), FXa, FIXa, and thrombin (17). Liaw's group showed that DOX induces a procoagulant phenotype in human endothelial cells (ECs) by increasing the PS flip to the cell surface which enhances activity of preexisting tissue factor (TF), without increasing its expression level (16). Interestingly, this effect was not seen for methotrexate nor 5-fluorouracil treated ECs (16). Further, the increase in surface PS on the ECs was associated with DOX-induced EC apoptosis (16). Later, Boles et al. (15) confirmed that the anthracycline daunorubicin also increased cellular TF activity without affecting TF protein levels, but rather by enhancing PS surface exposure on the human monocytic cell line THP-1 (Figure 1). DOX had a similar effect on platelets, causing increased PS surface exposure due to apoptotic pathway activation in DOX-exposed human platelets and subsequently resulting in enhanced procoagulant activity (14). The authors linked the increased PS exposure to DOX-induced platelet mitochondrial dysfunction at doses of 2.5–7.5 mg/kg in rats (13). Interestingly, at a cardiotoxic DOX dose of 25 mg/kg apoptosis-dependent thrombocytopenia was observed as early as 4 h after DOX injection in rats (13).

Moreover, daunorubicin was shown to increase the release of TF+ extracellular vesicles (EV) from THP-1 cells *in vitro* (Figure 1) (15). Increased anthracycline-induced EV release was confirmed by others (18–20). DOX-induced EVs are enriched for 4-hydroxy-2-nonenal (4-HNE), a marker for oxidative stress (19). 4-HNE can directly induce the release of TF+EVs from perivascular cells which can contribute to a prothrombotic state (21, 22). In line with this observation, TF+EVs were shown to enhance thrombus formation in multiple murine models of cancer-associated thrombosis (23, 24). Aside from its procoagulant effects, DOX is known to negatively affect the anticoagulant properties of ECs by downregulating the expression of the endothelial protein C receptor, leading to decreased protein C pathway activation (25).

## Effects on Blood Flow and Thrombus Formation *in vivo*

Injection of DOX (8 mg/kg) leads to occlusive vasoconstriction of smaller vessels (<15 μm) and vascular leakage in the murine femoral microvasculature within 4 min (26). Moreover, the same dose of DOX also reduces the blood flow in testicular arteries in mice within 15 min of injection (27). The authors linked these phenomena to DOX-induced vascular toxicity leading to EC-platelet interactions and the formation of EC-bound platelet microthrombi (27). Blood flow was restored by pre-treatment with low molecular weight heparin or the anti-platelet drug eptifibatide, suggesting that anti-platelet/anti-coagulant agents might be effective in reducing the detrimental vascular effects of DOX (27). DOX doses up to 7.5 mg/kg significantly enhanced thrombus sizes in a modified rat FeCl_3_ vena cava thrombosis model, without causing thrombocytopenia (14). In addition, in a vena cava stasis model DOX (7.5 mg/kg) caused increased thrombus formation that was reduced by administration of clopidogrel, aspirin or an inhibitor of platelet activated factor (28). These findings strongly suggest that DOX-induced venous thrombosis is dependent upon platelet activation (28).

## Coagulation-Dependent Signaling in Anthracycline-Induced Cardiotoxicity

While coagulation activation leads to fibrin deposition, the coagulation proteases that are generated in the process also lead to cleavage of protease-activated receptors (PARs) (29). PAR1 and PAR4 are activated by thrombin and are expressed on human platelets; their cleavage is the strongest platelet-activating stimulus. PAR3 also is activated by thrombin, but PAR3 mostly acts as co-factor for PAR4 and has only limited signaling function in humans (30). PAR2 is rather thrombin-insensitive and is primarily activated by the TF:FVIIa complex or FXa (31). Though PARs frequently are considered for their roles in platelets, they also are expressed on cardiomyocytes, where they contribute to the cardiac response to multiple injury models (29, 31, 32). The absence of PAR1 and PAR2 reduced infarct size and adverse cardiac remodeling in experimental heart failure (29, 31, 32). PAR4 activation can be cardioprotective or detrimental dependent on the chosen injury model and time point analyzed (31, 33–36).

With regard to chemotherapy-induced toxicity, PAR1 deficiency and PAR1 inhibition with the FDA-approved drug vorapaxar protected against DOX cardiotoxicity in mice (37). PAR1 activation exacerbated mitochondrial dysfunction and apoptosis in cardiac cells exposed to DOX *in vitro* (37). PAR1 deficiency was associated with reduced oxidative stress and apoptosis as well as decreased circulating cardiac troponin I and improved cardiac contractile function in the hearts of mice treated with 20 mg/kg DOX (37). PAR1 deficiency was also protective in a chronic DOX cardiotoxicity model (5 mg/kg/week for 5 weeks) (37). In line with these observations, PAR1 inhibition with the PAR1 inhibitor Q94 reduced toxic renal effects of DOX (15 mg/kg) in mice (38). Whether PAR2 or PAR4 contribute to DOX cardiotoxicity is the objective of ongoing investigations. Interestingly, PAR2 inhibition with FSLLRY-NH2 reduced nephropathy in a chronic rat DOX kidney injury model (1 mg/kg/day for 6 weeks) suggesting that PAR2 deficiency/inhibition might also be cardioprotective during DOX chemotherapy (39).

## Anthracyclines Induce Myocardial Atrophy

**Figure 2:**
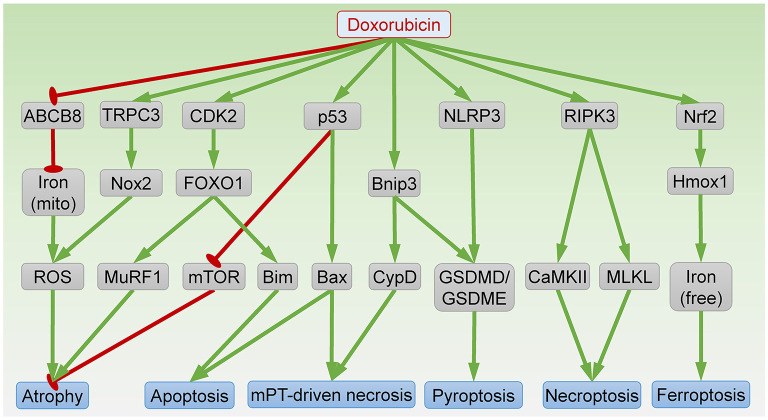
Signaling pathways in DOX-induced cardiomyocyte atrophy and death. ABCB8, ATP-binding cassette protein-B8; CaMKII, Ca^2+^-calmodulin–dependent protein kinase; CDK2, cyclin-dependent kinase 2; CypD, cyclophilin D; FOXO1, forkhead box O1; GSDMD/GSDME, gasdermin D/E; Hmox1, heme oxygenase-1; mito, mitochondria; MLKL, mixed lineage kinase domain like pseudokinase; mPT, mitochondrial permeability transition; mTOR, mammalian target of rapamycin; MuRF1, muscle RING finger 1; Nox2, NADPH oxidase 2; RIPK3, receptor-interacting protein kinase 3; ROS, reactive oxygen species; NLRP3, NLR family pyrin domain containing 3; TRPC3, transient receptor potential canonical 3. Arrows indicate activation; bar-headed lines indicate inhibition.

Anthracycline-based chemotherapies are known to cause abnormalities in heart morphology in cancer patients. Childhood cancer survivors who received anthracycline treatment have reduced ventricular wall thickness and myocardial mass later in life (40, 41). Recent evidence suggests that anthracyclines also cause a reduction in left ventricular mass in adult cancer patients (42–44). Importantly, an early decline in heart mass is associated with worse heart failure outcomes, emphasizing the importance of this phenomenon (42). A decrease in heart mass can be caused by reduced cardiomyocyte size (atrophy) and/or number (i.e., loss of cardiomyocytes due to cell death). Here, we summarize recently identified mechanisms underlying anthracycline-induced atrophy and cell death (Figure 2).

Similar to the clinical findings, exposure to the anthracycline DOX also reduces heart weight in mice (44–46). At the molecular level, DOX induces p53 expression, which is necessary for inactivation of mammalian target of rapamycin (mTOR), a serine-threonine kinase essential for protein synthesis (46). Interestingly, DOX-induced reductions in heart weight and myocyte size are abolished by cardiac-specific expression of dominant-interfering p53 or constitutively active mTOR, suggesting that DOX induces cardiac atrophy through p53-dependent inhibition of mTOR (46). Activation of mTOR by vascular endothelial growth factor-B (VEGF-B) gene therapy also prevents DOX-induced cardiac atrophy (47). Conversely, inducible ablation of mTOR in adult heart is sufficient to reduce cardiomyocyte size within 1–2 weeks (48). Taken together, these data indicate that mTOR inhibition is an important mechanism underlying DOX-induced atrophy.

DOX also induces expression of muscle RING finger 1 (MuRF1), a striated muscle-specific ubiquitin ligase and a key mediator of cardiac atrophy (44, 45). Mice lacking MuRF1 are resistant to DOX-induced reduction in heart mass, suggesting that MuRF1 is necessary for DOX-induced atrophy (44). Mechanistically, DOX exposure induces cyclin-dependent kinase 2 (CDK2)-mediated phosphorylation of forkhead box O1 (FOXO1) at Ser 249, resulting in FOXO1 activation and transcription of MuRF1 (45). Treatment with a FOXO1 inhibitor prevents DOX-induced cardiac atrophy and dysfunction (45). Collectively, FOXO1-dependent MuRF1 expression mediates DOX-induced atrophy.

Cardiac atrophy can occur as a result of oxidative stress. DOX exposure induces reactive oxygen species (ROS) generation through mitochondrial iron accumulation, owing to repression of ATP-binding cassette protein-B8 (ABCB8)-mediated mitochondrial iron export (49). Cardiac-specific ABCB8 transgenic mice are protected from DOX-induced ROS generation and atrophy (49). In addition, DOX exposure induces transient receptor potential canonical 3 (TRPC3)-dependent upregulation of NADPH oxidase 2 (Nox2) (50). Formation of the TRPC3-Nox2 complex amplifies ROS production and results in cardiac atrophy. Knockdown of TRPC3 or pharmacologic inhibition of TRPC3-Nox2 interaction attenuates DOX-induced atrophy in neonatal rat cardiomyocytes (NRCMs) (50). Moreover, mice lacking Nox2 are also resistant to DOX-induced cardiac atrophy (51). These findings suggest that enhanced ROS production resulting from mitochondrial iron accumulation or TRPC3-Nox2 complex formation also contributes to DOX-induced atrophy.

## Contributions of Programmed Cell Death to Anthracycline Cardiotoxicity

Exposure to anthracyclines triggers a variety of cell death modalities in the heart, resulting in cardiac cell loss. Anthracycline-induced cell death pathways have been reviewed in detail quite recently (52). A brief summary of the novel mechanisms of anthracycline-induced cardiomyocyte death is provided below.

### Apoptosis

Apoptosis is undoubtedly the most intensively studied form of cell death in anthracycline cardiotoxicity. DOX targets topoisomerase-IIβ to cause DNA double-strand breaks and initiate the intrinsic apoptosis pathway (53). DNA damage induces p53-dependent oligomerization of the Bcl2 family members Bak and Bax, which forms a pore in the outer mitochondrial membrane, resulting in cytochrome *c* release, caspase activation, and apoptosis. Accordingly, pharmacological inhibition of p53 or Bax blocks apoptosis and prevents DOX-induced cardiomyopathy (54, 55). It is noteworthy that p53 plays complicated roles in DOX-induced cardiotoxicity by modulating apoptosis-independent processes including mitochondrial biogenesis (56) and clonal hematopoiesis (57), as well as atrophy (46). In addition to the pore-forming effectors Bak and Bax, the pro-apoptotic Bcl2 family proteins also include activators (Bim, Bid, and Puma) that directly interact with the effectors to trigger apoptosis (58). DOX induces expression of Bim through CDK2-dependent FOXO1 activation (45, 59). Inhibition of either CDK2 or FOXO1 attenuates DOX-induced apoptosis and cardiac dysfunction (45, 59). Young age, a major risk factor for anthracycline cardiotoxicity in humans, is associated with higher sensitivity to apoptosis, further supporting an important role of apoptosis in anthracycline-related cardiotoxicity (60).

### Mitochondrial Permeability Transition Pore (mPTP)-Driven Necrosis

Necrosis driven by opening of the mPTP is characterized by rapid loss of the inner mitochondrial membrane potential and is dependent on cyclophilin D (CypD) (61). Recent evidence suggests that DOX treatment provokes mPTP-driven necrosis in cardiomyocytes (62). Mechanistically, DOX induces expression of Bnip3, which binds CypD to trigger mPTP opening and resultant necrosis (62). Bnip3 null mice are protected from DOX-induced mitochondrial damage, necrosis, and cardiac dysfunction (63). In addition, Bax and Bak are necessary for mPTP-driven necrosis (64, 65). Indeed, a small-molecule Bax inhibitor protects against DOX-induced necrosis *in vivo* (55).

### Necroptosis

Necroptosis is programmed cell necrosis that is initiated by binding of a death ligand (typically from the TNF superfamily) to a death receptor (such as Fas, TNFR1, or TRAIL) and culminates in plasma membrane permeabilization mediated by mixed lineage kinase domain like pseudokinase (MLKL) (61). MLKL activation and plasma membrane translocation requires phosphorylation by receptor-interacting protein kinase 3 (RIPK3) (66). DOX exposure upregulates cardiac RIPK3 and MLKL *in vivo* and *in vitro* to induce necroptosis (67). RIPK3 knockout mice are resistant to DOX-induced myocardial necrosis, cardiomyopathy and death (68). In this context, RIPK3 induces activation of Ca^2+^-calmodulin–dependent protein kinase (CaMKII) to trigger necroptosis (68). Moreover, DOX-induced cardiomyocyte death is blocked by the necroptosis inhibitor necrostatin-1, suggesting that necroptosis contributes to DOX-induced cardiomyocyte injury (67).

### Ferroptosis

Ferroptosis is a form of programmed cell death associated with mitochondrial damage owing to iron accumulation and lipid peroxidation (61). DOX induces nuclear factor erythroid 2–related factor 2 (Nrf2)-dependent transcription of heme oxygenase-1 (Hmox1) to trigger heme degradation, resulting in free iron accumulation, and ferroptosis (69). Treatment with the Hmox1 antagonist zinc protoporphyrin IX, the iron chelator dexrazoxane, or the ferroptosis inhibitor ferrostatin-1 protects against DOX-induced cardiomyopathy (69). Interestingly, loss of the E3 ubiquitin ligase tripartite motif containing-21 (TRIM21) enhances Nrf2 antioxidant activity but downregulates Hmox1, resulting in reduced ferroptosis and cardiotoxicity following DOX exposure (70). In addition, DOX reduces the levels of glutathione peroxidase 4 (GPx4), acyl-CoA thioesterase 1 (Acot1), and mitochondrial ubiquitin ligase MITOL, all of which augment lipid peroxidation and ferroptosis, in mouse heart (71–73).

### Pyroptosis

The major characteristic of pyroptosis is plasma membrane permeabilization mediated by gasdermin proteins such as gasdermin D (GSDMD) and gasdermin E (GSDME) (61). Cleavage of GSDMD by caspases 1, 3, 4, 5 or 11 results in GSDMD pore formation at the plasma membrane and subsequent pyroptosis. Pyroptosis is often pro-inflammatory owing to secretion of interleukin-1β and interleukin-18. DOX exposure induces cardiomyocyte pyroptosis *in vivo* and *in vitro* through NLR family pyrin domain containing 3 (NLRP3)-dependent activation of caspases 1, 3, and 11 (74, 75). In addition, Bnip3-dependent activation of caspase 3 also contributes to DOX-induced pyroptosis in cardiomyocytes (76).

## Conclusions

Here, we have reviewed our emerging understanding of the contributions of thrombosis, myocardial atrophy, and programmed cell death to the complex and multifaceted pathobiology of anthracycline-induced cardiovascular toxicity. Future work in our labs and others will further explicate the importance of these processes to anthracycline-induced cardiovascular toxicity and define whether they could represent novel therapeutic targets for prevention or treatment of these dose-limiting and potentially life-threatening adverse effects.

## Author Contributions

All authors drafted, edited, and approved the final version of the manuscript.

## Funding

The authors acknowledge the following funding support: SA: R01HL148432; ZC: R00HL119605, R56HL145034, R01HL151472; BCJ: R01HL140067.

## Conflict of Interest

The authors declare that the research was conducted in the absence of any commercial or financial relationships that could be construed as a potential conflict of interest.

## Publisher's Note

All claims expressed in this article are solely those of the authors and do not necessarily represent those of their affiliated organizations, or those of the publisher, the editors and the reviewers. Any product that may be evaluated in this article, or claim that may be made by its manufacturer, is not guaranteed or endorsed by the publisher.

## References

[1] Olson RD Boerth RC Gerber JG Nies AS. Mechanism of adriamycin cardiotoxicity: evidence for oxidative stress. Life Sci. (1981) 29:1393–401. 10.1016/0024-3205(81)90001-1 7029182

[2] Wallace KB Sardao VA Oliveira PJ. Mitochondrial determinants of doxorubicin-induced cardiomyopathy. Circ Res. (2020) 126:926–41. 10.1161/CIRCRESAHA.119.314681 32213135 PMC7121924

[3] Grover SP Hisada YM Kasthuri RS Reeves BN Mackman N. Cancer therapy-associated thrombosis. Arterioscler Thromb Vasc Biol. (2021) 41:1291–305. 10.1161/ATVBAHA.120.314378 33567864 PMC7990713

[4] Weiss RB Tormey DC Holland JF Weinberg VE. Venous thrombosis during multimodal treatment of primary breast carcinoma. Cancer Treat Rep. (1981) 65:677–9. 7248984

[5] Goodnough LT Saito H Manni A Jones PK Pearson OH. Increased incidence of thromboembolism in stage IV breast cancer patients treated with a five-drug chemotherapy regimen. A study of 159 patients. Cancer. (1984) 54:1264–8. 10.1002/1097-0142(19841001)54:7<1264::AID-CNCR2820540706>3.0.CO;2-R 6547874

[6] Zangari M Siegel E Barlogie B Anaissie E Saghafifar F Fassas A. Thrombogenic activity of doxorubicin in myeloma patients receiving thalidomide: implications for therapy. Blood. (2002) 100:1168–71. 10.1182/blood-2002-01-0335 12149193

[7] Nolan L Darby A Boleti K Simmonds P. The incidence of symptomatic thromboembolism in patients receiving adjuvant anthracycline-based chemotherapy for early stage breast cancer. Breast. (2011) 20:151–4. 10.1016/j.breast.2010.09.001 20970333

[8] Dentali F Squizzato A Ageno W. The metabolic syndrome as a risk factor for venous and arterial thrombosis. Semin Thromb Hemost. (2009) 35:451–7. 10.1055/s-0029-1234140 19739035

[9] Gomez-Sanchez EP. Metabolic syndrome: synergistic risks for doxorubicin-induced cardiotoxicity. J Cardiovasc Pharmacol. (2021) 78:782–3. 10.1097/FJC.0000000000001140 34581695 PMC8665042

[10] Guenancia C Lefebvre A Cardinale D Yu AF Ladoire S Ghiringhelli F. Obesity as a risk factor for anthracyclines and trastuzumab cardiotoxicity in breast cancer: a systematic review and meta-analysis. J Clin Oncol. (2016) 34:3157–65. 10.1200/JCO.2016.67.4846 27458291 PMC5569689

[11] L'Abbate S Russo I Kusmic C. The role of metabolic diseases in cardiotoxicity associated with cancer therapy: what we know, what we would know. Life Sci. (2020) 255:117843. 10.1016/j.lfs.2020.117843 32464123

[12] Russo M Della Sala A Tocchetti CG Porporato PE Ghigo A. Metabolic aspects of anthracycline cardiotoxicity. Curr Treat Options Oncol. (2021) 22:18. 10.1007/s11864-020-00812-1 33547494 PMC7864817

[13] Kim EJ Lim KM Kim KY Bae ON Noh JY Chung SM. Doxorubicin-induced platelet cytotoxicity: a new contributory factor for doxorubicin-mediated thrombocytopenia. J Thromb Haemost. (2009) 7:1172–83. 10.1111/j.1538-7836.2009.03477.x 19426282

[14] Kim SH Lim KM Noh JY Kim K Kang S Chang YK. Doxorubicin-induced platelet procoagulant activities: an important clue for chemotherapy-associated thrombosis. Toxicol Sci. (2011) 124:215–24. 10.1093/toxsci/kfr222 21865289

[15] Boles JC Williams JC Hollingsworth RM Wang JG Glover SL Owens AP 3rd. Anthracycline treatment of the human monocytic leukemia cell line THP-1 increases phosphatidylserine exposure and tissue factor activity. Thromb Res. (2012) 129:197–203. 10.1016/j.thromres.2011.06.022 21762960

[16] Swystun LL Shin LY Beaudin S Liaw PC. Chemotherapeutic agents doxorubicin and epirubicin induce a procoagulant phenotype on endothelial cells and blood monocytes. J Thromb Haemost. (2009) 7:619–26. 10.1111/j.1538-7836.2009.03300.x 19187077

[17] Dahlback B Villoutreix BO. Regulation of blood coagulation by the protein C anticoagulant pathway: novel insights into structure-function relationships and molecular recognition. Arterioscler Thromb Vasc Biol. (2005) 25:1311–20. 10.1161/01.ATV.0000168421.13467.82 15860736

[18] Aubertin K Silva AK Luciani N Espinosa A Djemat A Charue D. Massive release of extracellular vesicles from cancer cells after photodynamic treatment or chemotherapy. Sci Rep. (2016) 6:35376. 10.1038/srep35376 27752092 PMC5067517

[19] Yarana C Carroll D Chen J Chaiswing L Zhao Y Noel T. Extracellular vesicles released by cardiomyocytes in a doxorubicin-induced cardiac injury mouse model contain protein biomarkers of early cardiac injury. Clin Cancer Res. (2018) 24:1644–53. 10.1158/1078-0432.CCR-17-2046 29070527 PMC6193451

[20] Zhang C Yang Z Zhou P Yu M Li B Liu Y. Phosphatidylserine-exposing tumor-derived microparticles exacerbate coagulation and cancer cell transendothelial migration in triple-negative breast cancer. Theranostics. (2021) 11:6445–60. 10.7150/thno.53637 33995667 PMC8120203

[21] Ansari SA Keshava S Pendurthi UR Rao LVM. Oxidative stress product, 4-hydroxy-2-nonenal, induces the release of tissue factor-positive microvesicles from perivascular cells into circulation. Arterioscler Thromb Vasc Biol. (2021) 41:250–65. 10.1161/ATVBAHA.120.315187 33028097 PMC7752210

[22] Antoniak S Mackman N. New cellular source of TF (tissue factor)-positive extracellular vesicles in the circulation. Arterioscler Thromb Vasc Biol. (2021) 41:266–8. 10.1161/ATVBAHA.120.315437 33356372 PMC7773051

[23] Hisada Y Mackman N. Update from the laboratory: mechanistic studies of pathways of cancer-associated venous thrombosis using mouse models. Hematology Am Soc Hematol Educ Program. (2019) 2019:182–6. 10.1182/hematology.2019000025 31808871 PMC6913477

[24] Hisada Y Mackman N. Cancer cell-derived tissue factor-positive extracellular vesicles: biomarkers of thrombosis and survival. Curr Opin Hematol. (2019) 26:349–56. 10.1097/MOH.0000000000000521 31261175 PMC6677240

[25] Woodley-Cook J Shin LY Swystun L Caruso S Beaudin S Liaw PC. Effects of the chemotherapeutic agent doxorubicin on the protein C anticoagulant pathway. Mol Cancer Ther. (2006) 5:3303–11. 10.1158/1535-7163.MCT-06-0154 17172434

[26] Bar-Joseph H Stemmer SM Tsarfaty I Shalgi R Ben-Aharon I. Chemotherapy-induced vascular toxicity–real-time in vivo imaging of vessel impairment. J Vis Exp. (2015) 2015:e51650. 10.3791/51650 25590564 PMC4354501

[27] Aharon B Bar Joseph H Tzabari M Shenkman B Farzam N Levi M. Doxorubicin-induced vascular toxicity–targeting potential pathways may reduce procoagulant activity. PLoS ONE. (2013) 8:e75157. 10.1371/journal.pone.0075157 24073244 PMC3779248

[28] Bernat A Herbert JM. Effect of various drugs on adriamycin-enhanced venous thrombosis in the rat: importance of PAF. Thromb Res. (1994) 75:91–7. 10.1016/0049-3848(94)90143-0 8073411

[29] Antoniak S Mackman N. Coagulation, protease-activated receptors, viral myocarditis. J Cardiovasc Transl Res. (2014) 7:203–11. 10.1007/s12265-013-9515-7 24203054 PMC3943797

[30] Bretschneider E Spanbroek R Lotzer K Habenicht AJ Schror K. Evidence for functionally active protease-activated receptor-3 (PAR-3) in human vascular smooth muscle cells. Thromb Haemost. (2003) 90:704–9. 10.1160/TH03-04-0203 14515192

[31] Antoniak S Sparkenbaugh E Pawlinski R. Tissue factor, protease activated receptors and pathologic heart remodelling. Thromb Haemost. (2014) 112:893–900. 10.1160/th14-03-0243 25104210 PMC4382753

[32] Antoniak S Pawlinski R Mackman N. Protease-activated receptors and myocardial infarction. IUBMB Life. (2011) 63:383–9. 10.1002/iub.441 21438116 PMC3121912

[33] Kleeschulte S Jerrentrup J Gorski D Schmitt J Fender AC. Evidence for functional PAR-4 thrombin receptor expression in cardiac fibroblasts and its regulation by high glucose: PAR-4 in cardiac fibroblasts. Int J Cardiol. (2018) 252:163–6. 10.1016/j.ijcard.2017.10.019 29249425

[34] Kolpakov MA Rafiq K Guo X Hooshdaran B Wang T Vlasenko L. Protease-activated receptor 4 deficiency offers cardioprotection after acute ischemia reperfusion injury. J Mol Cell Cardiol. (2016) 90:21–9. 10.1016/j.yjmcc.2015.11.030 26643815 PMC5332160

[35] Strande JL Hsu A Su J Fu X Gross GJ Baker JE. Inhibiting protease-activated receptor 4 limits myocardial ischemia/reperfusion injury in rat hearts by unmasking adenosine signaling. J Pharmacol Exp Ther. (2008) 324:1045–54. 10.1124/jpet.107.133595 18055876 PMC2935083

[36] Kolpakov MA Guo X Rafiq K Vlasenko L Hooshdaran B Seqqat R. Loss of protease-activated receptor 4 prevents inflammation resolution and predisposes the heart to cardiac rupture after myocardial infarction. Circulation. (2020) 142:758–75. 10.1161/CIRCULATIONAHA.119.044340 32489148 PMC9341277

[37] Antoniak S Tatsumi K Schmedes CM Grover SP Pawlinski R Mackman N. Protease-activated receptor 1 activation enhances doxorubicin-induced cardiotoxicity. J Mol Cell Cardiol. (2018) 122:80–7. 10.1016/j.yjmcc.2018.08.008 30098988 PMC6173317

[38] Guan Y Nakano D Zhang Y Li L Liu W Nishida M. A protease-activated receptor-1 antagonist protects against podocyte injury in a mouse model of nephropathy. J Pharmacol Sci. (2017) 135:81–8. 10.1016/j.jphs.2017.09.002 29110957

[39] Wang Y He Y Wang M Lv P Liu J Wang J. Role of protease-activated receptor 2 in regulating focal segmental glomerulosclerosis. Cell Physiol Biochem. (2017) 41:1147–55. 10.1159/000464121 28245472

[40] Lipshultz SE Colan SD Gelber RD Perez-Atayde AR Sallan SE Sanders SP. Late cardiac effects of doxorubicin therapy for acute lymphoblastic leukemia in childhood. N Engl J Med. (1991) 324:808–15. 10.1056/NEJM199103213241205 1997853

[41] Lipshultz SE Lipsitz SR Sallan SE Dalton VM Mone SM Gelber RD. Chronic progressive cardiac dysfunction years after doxorubicin therapy for childhood acute lymphoblastic leukemia. J Clin Oncol. (2005) 23:2629–36. 10.1200/JCO.2005.12.121 15837978

[42] Jordan JH Castellino SM Melendez GC Klepin HD Ellis LR Lamar Z. Left ventricular mass change after anthracycline chemotherapy. Circ Heart Fail. (2018) 11:e004560. 10.1161/CIRCHEARTFAILURE.117.004560 29991488 PMC6729136

[43] Ferreira de Souza T Quinaglia ACST Osorio Costa F Shah R Neilan TG Velloso L. Anthracycline therapy is associated with cardiomyocyte atrophy and preclinical manifestations of heart disease. JACC Cardiovasc Imaging. (2018) 11:1045–55. 10.1016/j.jcmg.2018.05.012 30092965 PMC6196358

[44] Willis MS Parry TL Brown DI Mota RI Huang W Beak JY. Doxorubicin exposure causes subacute cardiac atrophy dependent on the striated muscle-specific ubiquitin ligase MuRF1. Circ Heart Fail. (2019) 12:e005234. 10.1161/CIRCHEARTFAILURE.118.005234 30871347 PMC6422170

[45] Xia P Chen J Liu Y Fletcher M Jensen BC Cheng Z. Doxorubicin induces cardiomyocyte apoptosis and atrophy through cyclin-dependent kinase 2-mediated activation of forkhead box O1. J Biol Chem. (2020) 295:4265–76. 10.1074/jbc.RA119.011571 32075913 PMC7105316

[46] Zhu W Soonpaa MH Chen H Shen W Payne RM Liechty EA. Acute doxorubicin cardiotoxicity is associated with p53-induced inhibition of the mammalian target of rapamycin pathway. Circulation. (2009) 119:99–106. 10.1161/CIRCULATIONAHA.108.799700 19103993 PMC2630181

[47] Rasanen M Degerman J Nissinen TA Miinalainen I Kerkela R Siltanen A. VEGF-B gene therapy inhibits doxorubicin-induced cardiotoxicity by endothelial protection. Proc Natl Acad Sci USA. (2016) 113:13144–9. 10.1073/pnas.1616168113 27799559 PMC5135329

[48] Zhang D Contu R Latronico MV Zhang J Rizzi R Catalucci D. MTORC1 regulates cardiac function and myocyte survival through 4E-BP1 inhibition in mice. J Clin Invest. (2010) 120:2805–16. 10.1172/JCI43008 20644257 PMC2912201

[49] Ichikawa Y Ghanefar M Bayeva M Wu R Khechaduri A Naga Prasad SV. Cardiotoxicity of doxorubicin is mediated through mitochondrial iron accumulation. J Clin Invest. (2014) 124:617–30. 10.1172/JCI72931 24382354 PMC3904631

[50] Shimauchi T Numaga-Tomita T Ito T Nishimura A Matsukane R Oda S. TRPC3-Nox2 complex mediates doxorubicin-induced myocardial atrophy. JCI Insight. (2017) 2:93358. 10.1172/jci.insight.93358 28768915 PMC5543921

[51] Zhao Y McLaughlin D Robinson E Harvey AP Hookham MB Shah AM. Nox2 NADPH oxidase promotes pathologic cardiac remodeling associated with Doxorubicin chemotherapy. Cancer Res. (2010) 70:9287–97. 10.1158/0008-5472.CAN-10-2664 20884632 PMC2984551

[52] Christidi E Brunham LR. Regulated cell death pathways in doxorubicin-induced cardiotoxicity. Cell Death Dis. (2021) 12:339. 10.1038/s41419-021-03614-x 33795647 PMC8017015

[53] Zhang S Liu X Bawa-Khalfe T Lu LS Lyu YL Liu LF. Identification of the molecular basis of doxorubicin-induced cardiotoxicity. Nat Med. (2012) 18:1639–42. 10.1038/nm.2919 23104132

[54] Saleme B Gurtu V Zhang Y Kinnaird A Boukouris AE Gopal K. Tissue-specific regulation of p53 by PKM2 is redox dependent and provides a therapeutic target for anthracycline-induced cardiotoxicity. Sci Transl Med. (2019) 11:aau8866. 10.1126/scitranslmed.aau8866 30728290

[55] Amgalan D Garner TP Pekson R Jia XF Yanamandala M Paulino V. A small-molecule allosteric inhibitor of BAX protects against doxorubicin-induced cardiomyopathy. Nat Cancer. (2020) 1:315–28. 10.1038/s43018-020-0039-1 32776015 PMC7413180

[56] Li J Wang PY Long NA Zhuang J Springer DA Zou J. p53 prevents doxorubicin cardiotoxicity independently of its prototypical tumor suppressor activities. Proc Natl Acad Sci USA. (2019) 116:19626–34. 10.1073/pnas.1904979116 31488712 PMC6765288

[57] Sano S Wang Y Ogawa H Horitani K Sano M Polizio AH. TP53-mediated therapy-related clonal hematopoiesis contributes to doxorubicin-induced cardiomyopathy by augmenting a neutrophil-mediated cytotoxic response. JCI Insight. (2021) 6:146076. 10.1172/jci.insight.146076 34236050 PMC8410064

[58] Chi X Nguyen D Pemberton JM Osterlund EJ Liu Q Brahmbhatt H. The carboxyl-terminal sequence of bim enables bax activation and killing of unprimed cells. Elife. (2020) 9:44525. 10.7554/eLife.44525 31976859 PMC6980855

[59] Xia P Liu Y Chen J Coates S Liu DX Cheng Z. Inhibition of cyclin-dependent kinase 2 protects against doxorubicin-induced cardiomyocyte apoptosis and cardiomyopathy. J Biol Chem. (2018) 293:19672–85. 10.1074/jbc.RA118.004673 30361442 PMC6314117

[60] Sarosiek KA Fraser C Muthalagu N Bhola PD Chang W McBrayer SK. Developmental regulation of mitochondrial apoptosis by c-Myc governs age- and tissue-specific sensitivity to cancer therapeutics. Cancer Cell. (2017) 31:142–56. 10.1016/j.ccell.2016.11.011 28017613 PMC5363285

[61] Galluzzi L Vitale I Aaronson SA Abrams JM Adam D Agostinis P. Molecular mechanisms of cell death: recommendations of the Nomenclature Committee on Cell Death 2018. Cell Death Differ. (2018) 25:486–541. 10.1038/s41418-017-0012-4 29362479 PMC5864239

[62] Dhingra R Guberman M Rabinovich-Nikitin I Gerstein J Margulets V Gang H. Impaired NF-kappaB signalling underlies cyclophilin D-mediated mitochondrial permeability transition pore opening in doxorubicin cardiomyopathy. Cardiovasc Res. (2020) 116:1161–74. 10.1093/cvr/cvz240 31566215 PMC7177490

[63] Dhingra R Margulets V Chowdhury SR Thliveris J Jassal D Fernyhough P. Bnip3 mediates doxorubicin-induced cardiac myocyte necrosis and mortality through changes in mitochondrial signaling. Proc Natl Acad Sci USA. (2014) 111:E5537–44. 10.1073/pnas.1414665111 25489073 PMC4280597

[64] Whelan RS Konstantinidis K Wei AC Chen Y Reyna DE Jha S. Bax regulates primary necrosis through mitochondrial dynamics. Proc Natl Acad Sci USA. (2012) 109:6566–71. 10.1073/pnas.1201608109 22493254 PMC3340068

[65] Karch J Kwong JQ Burr AR Sargent MA Elrod JW Peixoto PM. Bax and Bak function as the outer membrane component of the mitochondrial permeability pore in regulating necrotic cell death in mice. Elife. (2013) 2:e00772. 10.7554/eLife.00772 23991283 PMC3755340

[66] D.P. Del Re Amgalan D Linkermann A Liu Q Kitsis RN. Fundamental mechanisms of regulated cell death and implications for heart disease. Physiol Rev. (2019) 99:1765–817. 10.1152/physrev.00022.2018 31364924 PMC6890986

[67] Yu X Ruan Y Huang X Dou L Lan M Cui J. Dexrazoxane ameliorates doxorubicin-induced cardiotoxicity by inhibiting both apoptosis and necroptosis in cardiomyocytes. Biochem Biophys Res Commun. (2020) 523:140–6. 10.1016/j.bbrc.2019.12.027 31837803

[68] Zhang T Zhang Y Cui M Jin L Wang Y Lv F. CaMKII is a RIP3 substrate mediating ischemia- and oxidative stress-induced myocardial necroptosis. Nat Med. (2016) 22:175–82. 10.1038/nm.4017 26726877

[69] Fang X Wang H Han D Xie E Yang X Wei J. Ferroptosis as a target for protection against cardiomyopathy. Proc Natl Acad Sci USA. (2019) 116:2672–80. 10.1073/pnas.1821022116 30692261 PMC6377499

[70] Hou K Shen J Yan J Zhai C Zhang J Pan JA. Loss of TRIM21 alleviates cardiotoxicity by suppressing ferroptosis induced by the chemotherapeutic agent doxorubicin. EBioMedicine. (2021) 69:103456. 10.1016/j.ebiom.2021.103456 34233258 PMC8261003

[71] Tadokoro T Ikeda M Ide T Deguchi H Ikeda S Okabe K. Mitochondria-dependent ferroptosis plays a pivotal role in doxorubicin cardiotoxicity. JCI Insight. (2020) 5:132747. 10.1172/jci.insight.132747 32376803 PMC7253028

[72] Liu Y Zeng L Yang Y Chen C Wang D Wang H. Acyl-CoA thioesterase 1 prevents cardiomyocytes from Doxorubicin-induced ferroptosis via shaping the lipid composition. Cell Death Dis. (2020) 11:756. 10.1038/s41419-020-02948-2 32934217 PMC7492260

[73] Kitakata H Endo J Matsushima H Yamamoto S Ikura H Hirai A. MITOL/MARCH5 determines the susceptibility of cardiomyocytes to doxorubicin-induced ferroptosis by regulating GSH homeostasis. J Mol Cell Cardiol. (2021) 161:116–29. 10.1016/j.yjmcc.2021.08.006 34390730

[74] Meng L Lin H Zhang J Lin N Sun Z Gao F. Doxorubicin induces cardiomyocyte pyroptosis via the TINCR-mediated posttranscriptional stabilization of NLR family pyrin domain containing 3. J Mol Cell Cardiol. (2019) 136:15–26. 10.1016/j.yjmcc.2019.08.009 31445005

[75] Tavakoli Dargani Z Singla DK. Embryonic stem cell-derived exosomes inhibit doxorubicin-induced TLR4-NLRP3-mediated cell death-pyroptosis. Am J Physiol Heart Circ Physiol. (2019) 317:H460–71. 10.1152/ajpheart.00056.2019 31172809 PMC6732475

[76] Zheng X Zhong T Ma Y Wan X Qin A Yao B. Bnip3 mediates doxorubicin-induced cardiomyocyte pyroptosis via caspase-3/GSDME. Life Sci. (2020) 242:117186. 10.1016/j.lfs.2019.117186 31862454

